# Environmentally sustainable management practices support veterinary staff wellbeing

**DOI:** 10.3389/fvets.2025.1614496

**Published:** 2025-07-11

**Authors:** Danielle Scott, Lyn Davis, Colleen Duncan

**Affiliations:** College of Veterinary Medicine and Biomedical Sciences, Colorado State University, Fort Collins, CO, United States

**Keywords:** veterinary technician, staff retention, workplace wellbeing, sustainability, sustainable business, education, clinical sustainability

## Abstract

Veterinary technicians and assistants are integral to the daily operations of veterinary practices and have the potential to serve as key advocates for sustainability within the profession. This study aimed to assess veterinary support staff perspectives on the health impacts of climate change on companion animals, their perceived professional responsibilities in addressing climate-related health issues, and the availability of relevant educational resources. An electronic survey was distributed to veterinary staff across the United States through veterinary practices, AVMA-accredited technician programs, and technician-focused social media platforms. Findings indicate that technicians, technician students, and assistants overwhelmingly acknowledge the occurrence of climate change and its relevance to animal health. Respondents expressed a strong belief that veterinary staff should be informed about the health implications of climate change, identified a substantial gap in education on this topic, and demonstrated support for implementing sustainable practices within clinical settings. These results underscore the urgent need to integrate climate-related health education into veterinary support staff curricula and highlight the potential for sustainable initiatives to contribute to improved staff engagement and retention.

## 1 Introduction

Climate change is the most significant global health threat of the 21st century, motivating over 100 human-centered health care organizations to join forces and declare it a public health emergency (1, 2). Conversely, the veterinary field has been criticized for its lack of action, revealing the opportunity to advocate for animal health through policy engagement and climate mitigation efforts, including the adoption of environmentally sustainable practices in clinical settings (3, 4). These actions are desired by veterinary clients who overwhelmingly believe that climate change is relevant to animal health and expect their veterinarian to be knowledgeable on climate-induced health impacts their pet may be at risk for (4). Additionally, this action requires knowledge of such risks and resources to minimize environmental harm in the delivery of care. However, a recent review has highlighted a paucity of veterinary specific resources on this topic, while veterinarians and veterinary students report a lack of training and education available to them (5–8).

Veterinarians also report a number of ways in which climate change has impacted their own wellbeing, including blame for the role of animals in environmental change, and the responsibility of protecting animal health from the impacts associated with the changing climate (6). These feelings may contribute to compassion fatigue and burnout, a central consideration of the veterinary profession, leading to skilled individuals leaving the field (9, 10). Veterinary technicians are key members of the team, and the position requires formal education and rigorous training. Despite this investment, the turnover rate for veterinary technicians greatly exceeds that of the national average (11, 12). The implications of being short-staffed include paying for relief staff, low morale due to coworkers accumulating excess workload, diminished reputation from client perception, and even loss of business, thereby requiring a shift from emphasizing recruitment toward retention (13). Veterinary practices can harness longevity within support staff through the alignment of core values between employees and employers (12, 14–17), and environmentally sustainable business practices may be one way to approach common goals.

The perception of climate change as it relates to animal health has not been investigated in veterinary technicians and available education on this topic has not been assessed. However, comparisons may be drawn from the nursing field. Similar to nurses, veterinary technicians can continue to advance their education and specialize in specific disciplines, e.g., emergency and critical care, internal medicine, and more (18). There has been a call on the nursing profession to address climate change and although curriculum has been initiated in nursing programs, it has also been demonstrated that there is a need for ongoing education on climate induced health impacts and to define the nurse’s role in addressing the issue (18–20). We suspect a similar experience of veterinary technicians and support staff. The objective of this study was to explore veterinary technician views on the perceived health effects of climate change and pets, the role the veterinary profession has in addressing this topic, and educational opportunities on the topic.

## 2 Materials and methods

Veterinary technician, veterinary assistant, and veterinary technician student input was obtained through an online questionnaire. The link was distributed to AVMA accredited veterinary technician programs, as a link on social media websites targeting veterinary technician groups and to veterinary clinics around the United States utilizing AAHA Hospital Locator, Yelp, and Google searches to obtain email addresses. Ten raffled $50 gift certificates to Amazon were offered as an incentive to participate in the survey, with random.org used to randomize respondent selection for the certificate.

The survey was modeled after similar research projects involving veterinary students and veterinarians to facilitate comparisons across the field (4, 6, 7, 21). Questions (Supplementary material 1) were broadly categorized as: demographic information; perceptions of climate change as it relates to animal health; veterinary medicine’s role in addressing climate change; sustainable business practices in veterinary medicine; and lastly, educational opportunities available to support staff. There were four fill-in-the-blank, two select-all-that-apply, 14 Likert scale, 18 multiple choice responses, and one consent question to proceed with the survey. Respondents were required to be 18 years or older, certified veterinary technicians or the equivalent, i.e., CVT, LVT, or RVT, non-licensed technicians, and veterinary assistants, or veterinary technician students. Participants were anonymous. No question required a response to submit the survey and as not all questions were answered by each survey respondent, percentages were calculated out of total responses per question. Descriptive data analysis was conducted using commercially available software. The survey was classified as exempt by the Colorado State University Institutional Review Board. The survey was available from July 21 to August 21, 2020.

## 3 Results

A total of 619 veterinary staff members accessed the online survey and consented to participate. Those that gave consent but did not answer any further questions (n = 27) or answered only demographic questions, but did not complete any questions regarding climate change (n = 28) were also excluded from final analysis. Nineteen respondents failed to meet the inclusion criteria. The final analysis included 545 responses.

Certified veterinary technicians (CVT, LVT and RVTs) made up most of the respondents (46.6%, 254/545), followed by non-licensed technicians/veterinary assistants (33.8%, 184/545). Veterinary technician students comprised 19.6% of respondents (107/545). Most respondents (33.8%, 184/545) reported working in veterinary medicine for more than 10 years, followed by 3–10 years (31.9%, 174/545), then 1–3 years (21.1%, 115/545). The remaining respondents are full-time veterinary technician students (13.2%, 72/545).

Those responding as female comprised most responses (94.7%, 516/545). Those that identify as male represent 4.4% (24/545), and the remaining 0.7% stated their gender was not listed (4/545). One respondent did not answer this question (0.2%, 1/545). Most respondents were between the ages of 25–34 years old (36.9%, 201/545), followed by 18–24 years old (30.8%, 168/545), 35–44 years (17.3%, 94/545), 45–54 years (9.4%, 51/545), 55–64 years (5.1%, 28/545) and greater than 65 years old (0.6%, 3/545). Responses were received from every state except for Rhode Island and Wyoming. Most respondents (49.7%, 271/545) reported working in a suburban area, followed by 28.4% (155/545) working in an urban area. Those working in a rural area represented 16.0% (87/545) of respondents. Few respondents indicated they do not know the region in which they work (5.3%, 29/545) and 0.6% (3/545) indicated ‘other.’ Regarding political affiliation, 31.6% identify as moderate/middle of the road (172/545), followed by somewhat liberal (27.9%, 152/545), very liberal (18.4%, 100/545), somewhat conservative (12.7%, 69/545), and lastly 3.9% (21/545) identify as very conservative. The remaining respondents indicated ‘other’ for their political affiliation (5.7%, 31/545).

Level of education was asked and 35.4% (193/545) of respondents have achieved their associate’s degree, followed by those that achieved their bachelor’s degree (33.2%, 181/545). The third most represented education level was a high school graduate, diploma or equivalent (e.g., GED) (20.0%, 109/545). Those who completed a credential program (e.g., RVT/CVT/LVT, VTS, CVPM) comprised 5.5% (30/545) of respondents. Master’s degree holders represented 3.9% (21/545) followed by a doctorate degree (0.6%, 3/545). Those indicating ‘other’ encompassed 0.9% (5/545), two respondents did not answer this question (0.4%, 2/545) and one respondent has received no formal education (0.2%, 1/545).

### 3.1 Perceptions on climate change and pet health

**Figure 1:**
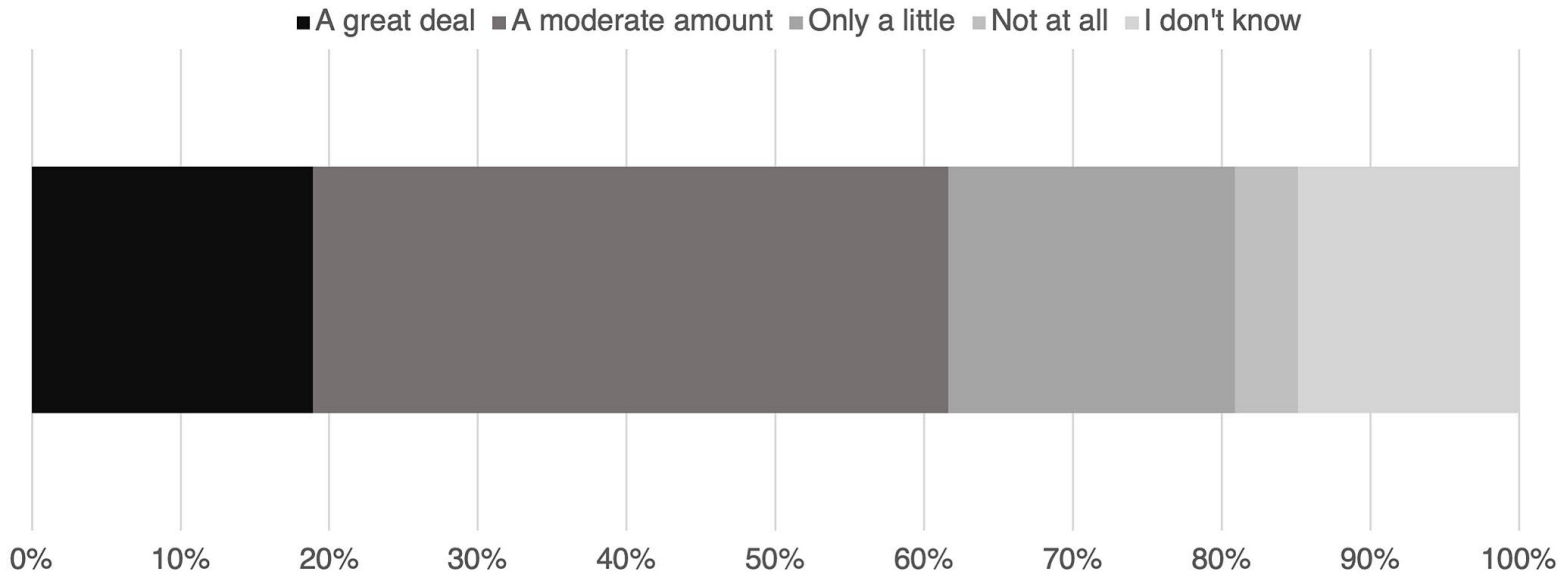
Responses (*n* = 545) to the question “how much, if at all, do you think climate change is relevant to direct patient care?”.

The overwhelming majority (94.0%, 512/545) believe climate change is occurring. Only 2.4% (13/545) do not believe climate change is occurring, and 3.7% (20/545) do not know if climate change is occurring. This survey asked how much, if at all, do you think climate change is relevant to direct veterinary patient care? The vast majority (80.9%, 441/545) believe that climate change has at least some relevance (Figure 1). When asked “how knowledgeable do you feel about the association between climate change and animal health impacts?” 42.4% (231/545) responded they were modestly knowledgeable, followed by 29.9% (163/545) feeling moderately knowledgeable. The next most common response was “not at all knowledgeable” with 20.6% (112/545) answering this way. Only 6.1% (33/545) feel very knowledgeable, and the remaining 1.1% (6/545) responded that this question is not applicable because climate change is not occurring.

**Figure 2:**
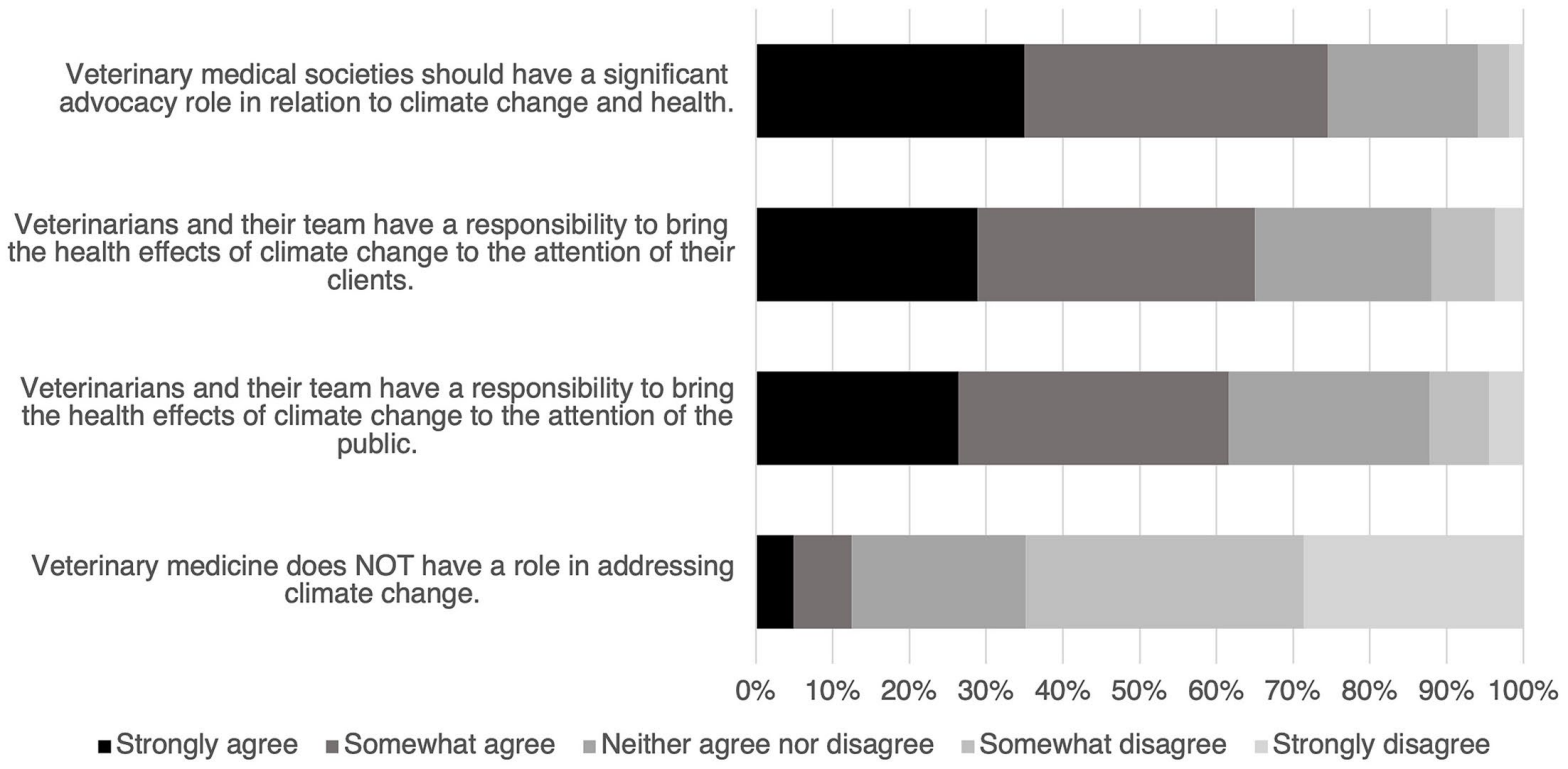
Level of agreement to statements on climate change in the survey. The number of respondents varied by statement; from top to bottom *n* = 491, 492, 492, 489.

The survey asked respondents to indicate their level of agreement to several statements (Figure 2) and most respondents agreed or strongly agreed that veterinary medical societies should have a significant advocacy role in relationship to climate change and health; veterinarians and their team have a responsibility to bring the health effects of climate change to the attention of their clients and the general public. When including a statement in the reverse, only 12.5% (61/489) agree that veterinary medicine does *not* have a role in addressing climate change.

Respondents were asked to select-all-that-apply about climate change and health topics that the veterinary community should be knowledgeable about (Table 1). Over 70% indicated that environmentally sustainable behaviors specific to veterinary medical practice (e.g., biomedical waste, building design), individual animal health impacts of climate change, and the economic impacts of climate change as related to animals, were topics the profession should be knowledgeable about. The survey also asked about how animals are currently being affected by climate change and how they will be affected in 10–20 years (Table 2). Over 61% of respondents believe that pets are currently being affected by multiple impacts and that percentage increases to over 67% in the future.

**Table 1:** Climate change health topics respondents believe the veterinary community should be knowledgeable about.

Climate change and health topic	Percent of total answering the question (*n* = 470)
Environmentally sustainable behaviors specific to veterinary medical practice (ex. biomedical waste, building design)	86.60%
Individual animal health impacts of climate change	80.85%
Economic impacts of climate change as related to animals (example: production animals)	73.83%
Personal actions to reduce environmental footprint (e.g., transportation, food choices, energy use, water use)	62.13%
Public (human) health impacts of climate change	55.11%
Research on the health impacts of climate change	47.66%
Policy and legislation relevant to climate change and health	36.38%
Social impacts of climate change	31.49%

**Table 2:** Respondents belief of how animals are currently being affected by climate change and how they will be affected in 10–20 years.

Climate change health effect	Currently			In the next 10–20 years		
Percent	Yes	No	Unsure	Yes	No	Unsure
Declining air quality	70.83% (323/456)	11.40% (52/456)	17.76% (81/456)	83.19% (282/339)	3.24% (11/339)	13.57% (46/339)
Increasing extreme weather events	80.44% (366/455)	10.77% (49/455)	8.79% (40/455)	85.80% (290/338)	4.44% (15/338)	9.76% (33/338)
Increasing vector-borne disease	78.60% (360/458)	5.68% (26/458)	15.72% (72/458)	79.23% (267/337)	2.97% (10/337)	17.80% (60/337)
Increasing water-associated illness/stress	69.45% (316/455)	8.35% (38/455)	22.20% (101/455)	78.53% (267/340)	3.53% (12/340)	17.94% (61/340)
Reduced food safety, quality and security	61.81% (280/453)	16.11% (73/453)	22.08% (100/453)	67.86% (228/336)	8.33% (28/336)	23.81% (80/336)
Increased heat associated illness/stress	84.03% (384/457)	7.88% (36/457)	8.10% (37/457)	86.94% (293/337)	4.15% (14/337)	8.90% (30/337)

### 3.2 Veterinary practices and sustainability

Respondents were in overwhelming agreement that veterinarians should have a leadership role in encouraging offices, clinics, and hospitals to be as environmentally sustainable as possible, with 53.1% strongly agreeing, 33.9% somewhat agreeing, 9.6% neither agreeing nor disagreeing, 2.7% somewhat disagreeing, and 0.8% strongly disagreeing. Most respondents indicated it is extremely important to work at a clinic that strives to be as environmentally sustainable as possible (36.1%, 164/454), followed by 34.4% indicating it is very important (156/454). Those that indicated moderately important comprised 20.3% (92/454). Lastly, 6.8% (31/454) and 2.4% (11/454) indicated ‘slightly important’ and ‘not at all important’, respectively. Level of agreement was similarly strong for the statement “working at an environmentally focused clinic would have a positive effect on my work experience,” with 76.4% (375/491) of respondents indicating that it would have a positive effect, 18.9% (93/491) neither agreed nor disagreed and 4.7% (23/491) indicated that it would not have a positive effect. To explore if sustainability measures are a potential motivator for support staff, respondents were asked the likelihood of accepting a job at a clinic with a sustainability focus, given all other factors, e.g., quality of medicine, are equivalent. The overwhelming majority (76.0%, 346/455) reported being more likely to accept the job at the sustainable practice. Those that reported being unlikely comprised 4.2% (19/455) answering this question. Finally, 19.8% (90/455) are neither likely nor unlikely to choose one clinic over the other given sustainability efforts.

**Figure 3:**
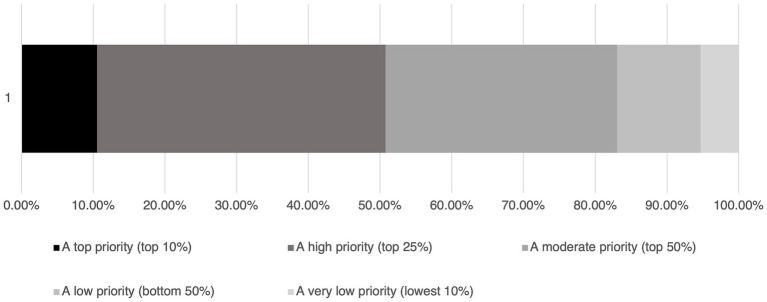
Responses (*n* = 455) to the question, “in your opinion, considering all issues facing the veterinary hospital you work at, how high of a priority should environmental sustainability be?”.

The survey asked, “considering all issues facing the veterinary hospital you work at, how high of a priority should environmental sustainability be?” and 83.1% believe it to be a priority (Figure 3). Respondents felt that informing clients that a veterinary clinic strives to be as environmentally sustainable as possible is important, with 25.6% (116/453) feeling it was extremely important, 35.3% (160/453) indicating it was very important and 27.4% (124/453) feeling it was moderately important. Only 8.6% (39/453) indicated it was slightly important, and 3.1% (14/453) did not feel it was important at all.

### 3.3 Climate change educational opportunities

There is consensus (77.1%, 386/494) that instruction on the environment, e.g., climate change, and its association with animal health should be integrated into veterinary technician education. The majority of veterinary technicians reported there are currently no opportunities to learn about health impacts associated with climate change (53.0%, 238/449) or they do not know if such opportunities exist (40.8%, 183/449). Only 6.2% (28/449) responded that opportunities for climate change education exist. The survey asked those who responded “yes” to identify where climate change education exists. Those that provided an answer indicated continuing education (*n* = 11), clubs (*n* = 6), electives (*n* = 5), and some indicated related curriculum was discussed in core classes (*n* = 5). The vast majority (74.8%, 336/449) of respondents indicated the need for opportunities to learn about the health impacts associated with climate change. The remaining 11.6% (52/449) did not see the need for such education, and 13.6% (61/449) did not know. When asked through what mechanism they felt education on health impacts from climate change should be provided, respondents were able to select all to a provided list. Of the 441 respondents answering this question, 72.8% (321/441) want to see this curriculum in continuing education opportunities, e.g., conferences and visiting lectures, and 49.7% (219/441) would like this offered through elective content in the veterinary technician curriculum. Including climate change curriculum as core content in the veterinary technician curriculum was desired by 43.5% (192/441) and 34.5% (152/441) would like student clubs and organizations to provide elective opportunities.

## 4 Discussion

Veterinary technicians, vet tech students, and veterinary assistants join their peers in recognizing that climate change is relevant to pet health, yet they largely lack education on this subject (6–8). Support staff believe that the veterinary field should be leaders addressing climate change, and sustainable business practices should be a priority. Seventy percent of veterinary technicians indicated that it is at least very important to work at an environmentally sustainable veterinary practice and over three-quarters of respondents expressed that working at such a clinic would have a positive effect on their work experience, choosing a position at the sustainable practice over a non-sustainable practice if all other aspects of the practice were equal. Our results support the need for veterinary-specific resources and education addressing climate change and animal health, including environmental sustainability in the delivery of veterinary care.

Building from previous studies exploring the veterinary profession (6, 7), our findings suggest that environmentally sustainable management practices could enhance veterinary technicians and assistants’ job satisfaction and wellbeing, thereby promoting staff retention and longevity. An ideology-infused psychological contract (IPC) can form between an employee and employer when values are shared between the two, leading to employee commitment, thereby benefiting the employer (14, 17). Our results reveal that an IPC contract is possible between veterinary practices and their support staff through the alignment of environmentally sustainable practices. Further, upward mobility within a position and holding a supervisory role has a positive association with job satisfaction (12). The introduction of a new job title, such as *Sustainability Coordinator* or *Sustainability Manager* could mutually benefit the employer and employee by providing a leadership role to a support staff with the goal of researching and implementing best environmental practices for the clinic.

In addition to bolstering best environmental practices within a clinics’ operations, support staff ought to be prepared for client discussions on the topic of climate change-associated health risk relevant to their pets. Such topics may include disaster preparedness, heat-associated illness, the spread of vector-borne infections, air quality, and more. However, our results join previous studies highlighting a need for additional climate change education both within and beyond the veterinary curriculum (6, 7). The vast majority of respondents (94%) reported there was no education available, or they were unaware of such education on this topic, despite 81% believing that climate change has at least some relevance to patient care. The lack of availability of climate change education is further demonstrated with only 6% of respondents feeling very knowledgeable regarding the animal health impacts of climate change. This study provides clear support contributing to the growing body of evidence that curriculum committees and continuing education organizers have a requirement to update their programs to incorporate climate change education for the veterinary profession.

This lack of available education on this topic is not unique to veterinary medicine. Although climate change health impacts are of great concern regarding human health, nurses also feel ill-equipped to address climate change within their profession (19). Veterinary technicians, much aligned with the nursing profession, play an important role in client education and recognize the responsibility of educating clients and the general public on the health effects of climate change. Those who are educated on this topic will be better equipped to keep their patients, their community, and themselves safe and healthy in a changing climate through fostering community resilience. Veterinary technicians who are interested can engage with organizers and request the incorporation of relevant material. Those who are particularly knowledgeable or trained are encouraged to submit abstracts at local, regional, and national conferences. Research has demonstrated the role of the nurse in addressing climate change includes policy making, nursing practice, and nursing education (19). The voice of the technician can also be amplified through engaging with governing bodies and encouraging the implementation of environmentally sustainable business practices within veterinary hospitals.

Limitations must be considered in the interpretation of our results. Due to the nature of the distribution of the questionnaire, this survey is best described as a convenience sample. While a database of AVMA accredited veterinary technician schools was utilized to distribute to veterinary technician students, we lacked a national database for working veterinary technicians and therefore also utilized AAHA Vet Finder, Yelp, and Google to obtain email addresses for veterinary clinics, which may have skewed responses. Despite this, responses were received from 48 out of 50 states, and the spread of responses received from urban, suburban and rural areas was similar to that of a survey of veterinarians on this topic (6). Study respondents were almost entirely women (94.7%), which compares to other studies surveying technicians and thus likely representative of the demographic of the veterinary profession’s technical staff (12, 22). Gender has been shown not to be a demographic influencer of climate change perceptions (4). Additionally, because this survey was conducted in 2019, it is possible that sentiments have shifted over time. However, Generation Z and Millennials are known to be the generations most concerned about climate change (23), and these generations comprised more than 65% of the study population. As Gen Z continues to enter the workforce, it is likely that pro-environmental attitudes will continue to grow. Collectively, this suggests that this sample from our study is likely representative of the technician workforce, although there may have been a response bias as those with an interest in this topic may have been more likely to participate.

Another potential limitation of the current study is that there are varying degrees of continuing education requirements among support staff. Our survey results include respondents self-described as registered/licensed/certified veterinary technician; non-licensed technician; veterinary assistant; veterinary technician student; and ‘other’ with the ability to fill in the answer. Only RVTs/LVTs/CVTs have a continuing education requirement for their licensure. Non-licensed positions may not know the educational opportunities available. To address this, we had respondents choose the option that best describes their role in veterinary medicine, in order to draw conclusions for the varying positions, e.g., RVT, assistant, technician student. To this degree, we can identify the availability, or lack thereof, of climate curriculum included in continuing education opportunities, as well as in veterinary technology programs. Lastly, it is important to note we refrained from using the term “veterinary nurses” in both our survey and reporting of results, as the term “nurse” is legally protected in many U.S. states (24). However, other countries may use “veterinary nurse” to describe an equivalent position of RVT/LVT/CVT and/or veterinary assistants. Additionally, educational requirements may differ depending on regulating agencies. Therefore, regional and international differences in title use and education requirements must be considered when generalizing results to regions outside the United States.

In conclusion, this study offers important insights into the perspectives of veterinary technicians, assistants, and students regarding climate change and its relevance to veterinary medicine. Findings highlight a strong belief among support staff in the profession’s responsibility to address climate-related animal health risks and to adopt environmentally sustainable business practices. These practices not only align with the values of many employees but may also contribute to improved job satisfaction and retention in a field challenged by high turnover. The results suggest that integrating sustainability efforts and climate change education into veterinary technician training and clinic operations could be mutually beneficial for staff wellbeing, patient care, and environmental responsibility. As in human healthcare, veterinary support staff are eager to engage with this issue but require institutional support, clear roles, and educational opportunities to do so effectively. Broadening curriculum offerings and creating sustainability focused roles in clinical settings represent meaningful next steps in empowering veterinary support staff to become key players in climate action within the profession.

## Data Availability

The original contributions presented in the study are included in the article/Supplementary material, further inquiries can be directed to the corresponding author.
