# Supplementary material for: Environmentally sustainable management practices support veterinary staff wellbeing
Source: Front Vet Sci. 2025 Jul 11;12:1614496. doi: 10.3389/fvets.2025.1614496 (PMC12289507; doi:10.3389/fvets.2025.1614496)
Supplement: Supplementary file 1 [file Table_1.docx]

# Supplementary Material 1: Veterinary Technician Survey Questions

Please choose the best option that describes your role in veterinary medicine:

- Registered/Licensed/Certified Veterinary Technician
- Non-licensed technician
- Veterinary Assistant
- Veterinary Technician Student
- Other

How long have you been working in veterinary medicine?

- 1-3 years
- 3-10 years
- >10 years
- I am currently a veterinary technician student In which state do you work in veterinary medicine?
- [drop down menu with all states and ‘other’] How old are you?
- 18-24 years
- 25-34 years
- 35-44 years
- 45-54 years
- 55-64 years
- >65 years

With which gender do you identify?

- Male
- Female
- Gender not listed
- Prefer not to answer

What is the highest degree or level of education you have completed. If currently enrolled, highest degree received.

- High School graduate, diploma or equivalent (e.g. GED)
- Associate's degree
- Bachelor's degree
- Master's degree
- Doctorate degree
- No formal education
- Credential Program (e.g. VTS, CVPM, etc)
- Other:

Which of the following best describes the region in which you work?

- Urban
- Suburban
- Rural
- Other, please list
- I don’t know

In general, do you think of yourself as:

- Very liberal
- Somewhat liberal
- Moderate, middle of the road
- Somewhat conservative
- Very conservative
- Other

Climate change refers to the idea that the world’s average temperature has been increasing over the past 150 years, may be increasing more in the future, and that the world’s climate is changing as a result. Do you think that climate change is happening at this time?

- Yes
- No
- I don’t know

How knowledgeable do you feel about the association between climate change and animal health impacts?

- Very knowledgeable
- Moderately knowledgeable
- Modestly knowledgeable
- Not at all knowledgeable
- Not applicable because climate change is not occurring

How much, if at all, do you think climate change is relevant to direct veterinary patient care?

- A great deal
- A moderate amount
- Only a little
- Not at all
- I don’t know

Please indicate your level of agreement with the following statements. [Table with response options for all statements of ‘Strongly agree, Somewhat agree, Neither agree nor disagree, Somewhat disagree, Strongly disagree’]

- Instruction on the environment (e.g., climate change) and its association with animal health impacts should be integrated into veterinary technician education.
- Veterinary medical societies should have a significant advocacy role in relation to climate change and health.
- I feel that actions I take in my personal and/or professional life can contribute to effective action on climate change.
- Veterinarians and their team have a responsibility to bring the health effects of climate change to the attention of the public.
- Veterinarians and their team have a responsibility to bring the health effects of climate change to the attention of their clients.
- Veterinarians should have a leadership role in encouraging offices, clinics, hospitals to be as environmentally sustainable as possible.
- Working at an environmentally focused clinic would have a positive effect on my work experience.
- Veterinary medicine does not have a role in addressing climate change.
- I desire a responsible disposal method for the plastic waste generated in a veterinary clinic.

Which of the following, if any, are climate change and health topics that the veterinary community should be knowledgeable about? (Select all that apply.)

- Individual animal health impacts of climate change
- Public (human) health impacts of climate change
- Economic impacts of climate change as related to animals (example: production animals)
- Social impacts of climate change
- Environmentally sustainable behaviors specific to veterinary medical practice (ex. biomedical waste, building design)
- Personal actions to reduce environmental footprint (e.g. transportation, food choices, energy use, water use)
- Policy and legislation relevant to climate change and health
- Research on the health impacts of climate change
- Other

In which of the following ways, if any, do you think veterinary patients are currently being affected by climate change or might be affected in the next 10-20 years? [Table with response options of ‘Yes, No, Unsure’ for both ‘Currently’ and ‘In the next 10-20 years’]

- Declining air quality
- Increasing extreme weather events
- Increasing vector-borne diseases
- Increasing water-associated illnesses/stress
- Reduced food safety, quality and security
- Increasing heat associated illness/stress
- Other, please list

How important do you think it is for clients to know that the veterinary clinic strives to be as environmentally sustainable as possible?

- Extremely important
- Very important
- Moderately important
- Slightly important
- Not at all important

How important is it for you that the veterinary clinic you work at strives to be as environmentally sustainable as possible?

- Extremely important
- Very important
- Moderately important
- Slightly important
- Not at all important

How likely are you to accept a job at a sustainably focused hospital over a non-sustainably focused hospital provided all other factors are equivalent (e.g. quality of medicine)?

- Extremely likely
- Somewhat likely
- Neither likely nor unlikely
- Somewhat unlikely
- Extremely unlikely

In your opinion, considering all issues facing the veterinary hospital you work at, how high of a priority should environmental sustainability be?

- A top priority (top 10%)
- A high priority (top 25%)
- A moderate priority (top 50%)
- A low priority (bottom 50%)
- A very low priority (lowest 10%)

Is there anything else you would like to add regarding sustainable business practices in veterinary medicine?

Within your veterinary technician education, are there currently opportunities to learn about health impacts associated with climate change?

- Yes, if so please explain (e.g. clubs, core classes, electives, continuing education, etc)
- No
- I don’t know

Within your veterinary technician education, do you see a need for opportunities to learn about health impacts associated with climate change?

- Yes
- No
- I don’t know

Through what mechanism do you believe education on health impacts from climate change should be provided? select all that apply

- Core content in the veterinary technician curriculum
- Elective content in the veterinary technician curriculum
- Elective opportunities through student clubs and organizations
- Continuing education opportunities (e.g. conferences, visiting lectures
- Other, please specify

Is there anything else you would like to add regarding climate change and education opportunities for veterinary technicians?
